# Effects of Inducible Nitric Oxide Synthase Inhibition on Cardiovascular Risk of Adult Endotoxemic Female Rats: Role of Estrogen

**DOI:** 10.3389/fphys.2018.01020

**Published:** 2018-07-31

**Authors:** Jaqueline C. Castardo-de-Paula, Blenda H. de Campos, Lorena de Jager, Eric D. T. Amorim, Nágela G. Zanluqui, Carine C. de Farias, Luciana Higachi, Phileno Pinge-Filho, Décio S. Barbosa, Marli C. Martins-Pinge

**Affiliations:** ^1^Department of Physiological Sciences, Center of Biological Sciences, State University of Londrina, Londrina, Brazil; ^2^Department of Pathological Sciences, Center of Biological Sciences, State University of Londrina, Londrina, Brazil; ^3^Department of Pathology, Clinical and Toxicological Analysis, Center of Health Sciences, University Hospital, State University of Londrina, Londrina, Brazil

**Keywords:** blood pressure, endotoxemia, heart rate, lipopolysaccharide, nitric oxide, ovariectomy

## Abstract

**Aim:** Autonomic modulation responds to ovarian hormones and estrogen increases nitric oxide bioavailability. Also, females have minor susceptibility to sepsis and a higher survival rate. However, few studies have evaluated the role of estrogen in cardiovascular, autonomic, and oxidative parameters during initial endotoxemia and under inducible nitric oxide synthase (iNOS) inhibition in female rats.

**Methods:** Female wistar rats were subjected to ovariectomy and divided into three groups: OVX (ovariectomized), OVX+E (OVX plus daily estradiol) and SHAM (false surgery). After 8 weeks, mean arterial pressure (MAP) and heart rate (HR) were recorded in non-anesthetized catheterized rats, before and after intravenous LPS injection, preceded by S-methylisothiourea sulfate (SMT) injection, or sterile saline. Cardiovascular recordings underwent spectral analysis for evaluation of autonomic modulation. Two hours after LPS, plasma was collected to assess total radical-trapping antioxidant (TRAP), nitrite levels (NO2), lipoperoxidation (LOOH), and paraoxonase 1 (PON1) activity.

**Results:** Two hours after LPS, females treated with SMT presented a decrease of MAP, when compared to saline-LPS groups. At this same time, all SMT+LPS groups presented an increase of sympathetic and a decrease of parasympathetic modulation of HR. Two hours after saline+LPS, OVX presented decreased total radical-trapping antioxidant (TRAP) compared to SHAM. When treated with SMT+LPS, OVX did not altered TRAP, while estradiol reduced LOOH levels.

**Conclusion:** iNOS would be responsible for sympathetic inhibition and consumption of antioxidant reserves of females during endotoxemia, since iNOS is inhibited, treatment with estradiol could be protective in inflammatory challenges.

## Introduction

Females of all ages have lower rates of infection and consequent mortality than males. This significant difference in inflammatory response between genders has long been noted and epidemiological and immunological evidence indicate female hormones as protagonists of these differences in the etiology and course of inflammatory processes (Straub, 2007). An example of the interaction between estradiol and inflammation are the sex differences that can be observed in sepsis (Angele et al., 2014). Sepsis and endotoxic shock are important medical complications with a high mortality rate caused by the endotoxin lipopolysaccharide (LPS), a component of gram-negative bacteria (Bone, 1992) used as an experimental tool for mimicking sepsis through the induction of endotoxemia. The body defends of LPS with a powerful sympathetic reflex leading to the suppression of the acute systemic inflammatory response, indicated by the reduction of TNF-α levels (Martelli et al., 2014). The key symptoms of shock are severe hypotension and vasoplegia, resulting in dysfunction of one or more vital organs (Cauwels, 2007).

The power spectral analysis is a non-invasive method to assess sympathovagal balance of heart rate variability (HRV) (Petrofsky et al., 2009). Alterations in HRV, which primarily reflect the tonic autonomic modulation, may have substantial clinical implications (Campos et al., 2014). Female rats had the most prominent high frequency (HF) component of spectral analysis, which represents the parasympathetic drive, during estrus compared with OVX and diestrus; the estrous cyclicity, as well as the cycle-related HF changes, disappeared with ovariectomy (OVX) (Kuo et al., 2010), corroborating estradiol’s participation in autonomic modulation. In humans, septic shock is characterized by reductions in HRV, LF, and LF: HF ratio, in addition to reducing systolic pressure variability and LFabs (Annane et al., 1999). Differently, in Sprague-Dawley rats of both sexes was observed an increase in HFnu, LFnu, and LF: HF ratio 2 h after LPS injection (Huang et al., 2010).

Losonczy et al. (2000) observed that females are resistant to the development of endotoxic shock by LPS. Hypotension is a major cause of death during shock, and nitric oxide (NO) is an important mediator. NO acts ambiguously during septic shock: the inhibition of its synthesis restores blood pressure, but inhibition of nitric oxide synthase (NOS) does not improve the progression of the condition. This discrepancy would be related to the isoforms, with endothelial NOS (eNOS) providing essential and protective NO and inducible NOS (iNOS) causing excessive vasodilation (Cauwels, 2007). iNOS is an calcium independent enzyme whose NO production control is produced during transcription and translation, since once activated iNOS produces NO until complete depletion of the substrate. LPS itself, among several pro-inflammatory mediators, is a stimulus to iNOS activity (Lirk et al., 2002). In the first few hours of LPS endotoxemia Losonczy et al. (2000) observed the induction of iNOS expression in the aorta and spleens of male rats, while females did not have detectable iNOS in the aorta. However, even resistant to the development of endotoxemic shock, females had higher NO levels after LPS, and OVX did not alter this parameter. Despite this, OVX female rodents exhibit higher morbidity and mortality after sepsis, further suggesting the protective capability of estradiol (Knoferl et al., 2002).

These data suggest the need for further investigations on the relation among iNOS, ovarian hormones, cardiovascular autonomic modulation and the protection of females against hypotension to LPS. The purpose of this study was to test the hypothesis that estrogen is the female hormone responsible for protection against hypotension in the first few hours of endotoxemia, and this protection would be related to the lower influence of iNOS on the cardiovascular system of the estrogen treated female rats.

## Materials and Methods

### Animals

Adult female Wistar rats were maintained in polypropylene cages, in a ventilated and controlled temperature chamber (22 ± 1°C) on a 12:12 h light-dark cycle with food (Nuvilab CR-1^®^; Nuvital, Colombo, Paraná, Brazil) and tap water available *ad libitum*. Rats were used only once and were acclimated to the testing room before the experiments, which was conducted during the light phase. At the end of experiments, rats were killed with an overdose of sodium tiopental (Thipentax^®^, Cristália, São Paulo, Brazil).

All experimental protocols were performed in accordance with the Ethical Guidelines of the State University of Londrina (UEL), process number 276.2013.81, Paraná, Brazil.

### Surgical Proceedings and Experimental Groups

**FIGURE 1:**
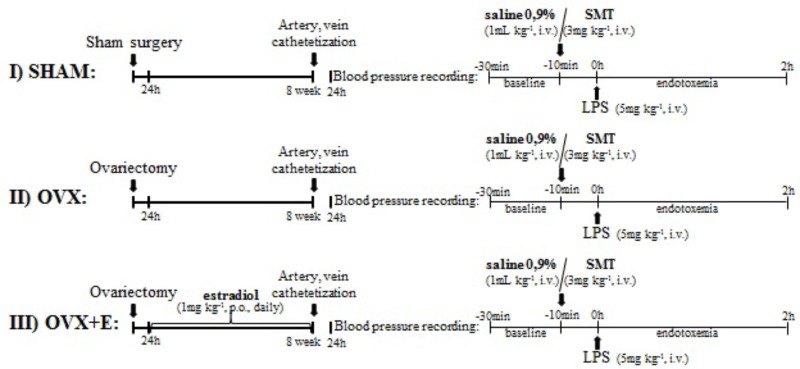
Experimental protocol. **(I)** and **(II)** sham or ovariectomized -operated rats were bred for 8 weeks and submitted to catheterization of femoral artery and vein. **(III)** OVX+E: OVX rats were treated with estradiol valerate (1 mg kg^-1^, gavage, once daily) for 8 weeks, beginning 24 h after ovariectomy, and submitted to catheterization of femoral artery and vein. 24 h after catheterization, blood pressure was recording for 10 min and saline (0.9%, i.v.) or SMT (3 mg kg^-1^, i.v.) was injected. After 10 min, LPS was injected (5 mg kg^-1^, i.v.) and blood pressure recording continued for 2 h. LPS, lipopolysaccharide; SMT, S-methylisothiourea.

Rats were subjected to bilateral ovariectomy or false surgery under ketamine and xylazine anaesthesia (100 and 6.7 mg kg^-1^, i.p.; Ceva Santé Animale, São Paulo, Brazil) and 24 h after were divided into experimental groups (*n* = 5–11, **Figure 1**):

SHAM: The procedure of sham operation consisted of anesthesia, visualization of the ovaries through incisions into the dorsal cavities, and closure of the wounds. These animals were kept in their boxes, without any intervention, for 8 weeks until the day of the experiment.
OVX: ovariectomy control group that received estradiol vehicle (almond oil, 0.5 mL kg^-1^, per day, p.o.; Generophlora drugs, Londrina, Paraná, Brazil);
OVX+E: OVX plus estradiol treatment group that received estradiol valerate (1 mg kg^-1^ per day, p.o.; Hangzhou Hetd Industry Co., Zhejiang, China) during 8 weeks (Ceylan-Isik et al., 2009).

Estrous cycle in the SHAM-operated females was monitored by microscopic investigation of the vaginal smear (Kauser et al., 1997) and only females in estrus phase were used in the experiments.

Eight weeks after the surgery and 24 h before the experiments, under ketamine–xylazine anesthesia, a polyethylene catheter was inserted into the femoral artery and vein and externalized dorsally with the purpose of monitoring blood pressure and administration of drugs. The catheter segments were constituted by welded segments of polyethylene PE-10 (4–5 cm) and polyethylene PE-50 (12–13 cm), which were filled with 0.9% saline and anticoagulant (15 U ml^-1^ heparin saline solution) and blocked with an occluder. After catheter implantation, they were exteriorized at the dorsal region subcutaneously and fixed to the skin by surgical suture. Following surgery, the animals returned to individual cages throughout the post-operative period. After 24 h, the baseline MAP and HR were recorded (da Cunha et al., 2014; Ariza et al., 2015; Castardo-de-Paula et al., 2017).

### Experimental Protocols

Represented in **Figure 1**.

#### Measurement of Baseline Cardiovascular Parameters

After 24 h of catheterization, the arterial cannula of the animal was attached to a pressure transducer (Powerlab model MLT0380) connected to a computerized recording system (Powerlab/ADInstruments), while the animal was awake and freely moving. The animals were kept in their individual cages and baseline recordings were obtained for at least 20 min before starting the protocol (da Cunha et al., 2014; Castardo-de-Paula et al., 2017).

#### LPS Induced Endotoxemia

After the basal recording, rats received by venous catheter a bolus injection of S-methylisothiourea (SMT) (3 mg kg^-1^, Santa Cruz Biotechnology^®^, Texas, TX, United States), a potent inhibitor of iNOS (Szabo et al., 1994; Su et al., 2007), or physiological saline 0.9% (1 mL kg^-1^), the SMT vehicle. After 10 min of recording of SMT or saline effects on blood pressure, a dose of 5 mg kg^-1^ of LPS of Escherichia coli (serotype 026:B6, Sigma Chemical Co.) (Mehanna et al., 2007) was administered as a bolus injection (i.v.). The cardiovascular effects of endotoxin were recorded for 2 h.

At the end of recording, plasma samples were collected for measurement of the nitrite and lipoperoxidation levels (LOOH), the plasma activity of paraoxonase 1 (PON1) and the total antioxidant capacity of plasma. A standard laboratory scale was used to measure body weight, tibia length and uterus weights (Gore et al., 2002; Voltera et al., 2008; Castardo-de-Paula et al., 2017).

### Biochemical Analysis

Plasmatic measurements of nitrite levels, lipoperoxidation, Total radical-trapping antioxidant parameter (TRAP), and Paraoxonase 1 (PON1) activity were performed as described and previously mentioned (Castardo-de-Paula et al., 2017).

### Heart Rate and Systolic Arterial Pressure Variability

The last 5 min of the recordings of arterial pressure, from pre-LPS treatment, and the last 3 min from 2 h post-LPS treatment, were processed using a specific computer program (LabChart 7 Pro^®^, ADInstruments, Bella Vista, NSW, Australia) capable of detecting inflection points in pressure pulses generating beat-by-beat time series of pulse interval (PI) and systolic arterial pressure (SAP). The frequency domain (PI and SAP variability) and power spectral analysis was performed using a custom software (CardioSeries^®^ v2.4) as previously described (Dutra et al., 2013; Castardo-de-Paula et al., 2017).

### Spontaneous Baroreflex Analysis

Baroreflex sensitivity (BRS) was assessed by the sequence method with the CardioSeries v2.4 computer program. The beat-to-beat time series of the PI and SAP values were used in the BRS analysis. The time series were analyzed for the sequences of four or more beats in which progressive increases in SAP were accompanied by progressive increases in PI or progressive reductions in SAP were accompanied by progressive reductions in PI. To detect the changes in SAP and PI, thresholds of 0 mmHg and 0 ms, respectively, were used. After detecting a ramp SAP (sequence of 4 or more beats in which progressive increases or reductions in SAP were or were not followed by increases or reductions in the PI), the computer program sought changes in PI without any interval, such as a delay of zero beats. A baroreflex sequence was used only when the correlation coefficient (r) between SAP and PI was ≥0.8. The BRS was determined from the slope of the linear regression between the SAP and PI of each baroreflex sequence (Ariza et al., 2015). The slope of the curve indicates the sensitivity or BRS gain.

### Statistical Analysis

An exploratory analysis was conducted to evaluate normal distribution (Shapiro–Wilk test) and homogeneity of variance (Levene test) of each variable. For variables that presented normal distribution and homogeneity of variance, parametric analysis were conducted. Morphometric parameters and area under de curves (AUC) were analyzed by 1way ANOVA followed by Bonferroni test. In order to achieve homogeneity in the data distribution, the percentage of changes in relation to time zero (0, moment of LPS injection) was established for the cardiovascular and autonomic parameters. Obtained graphs of cardiovascular, autonomic and biochemical data, for 2 h of endotoxemia, were analyzed with 2-way ANOVA and Bonferroni multiple comparisons test for *post hoc* analysis, the analyzed factors were surgery or hormone treatment (OVX, OVX+E, SHAM), pre-LPS injection (saline or SMT) and the surgery x pre-LPS factors interaction. For non-parametric data, multiple comparisons were performed using the Kruskal–Wallis followed by the Dunn test. In all cases, *P* ≤ 0.05 was considered as significant.

## Results

### Efficacy of Ovariectomy and Hormonal Treatment

**FIGURE 2:**
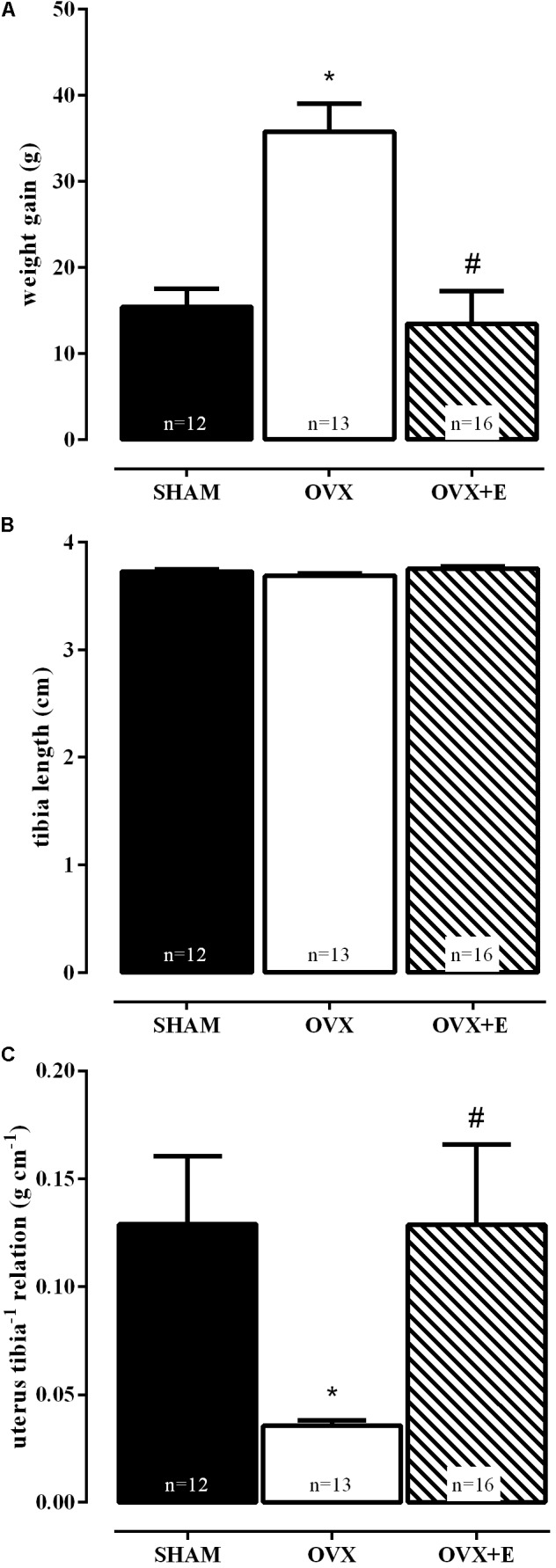
Effects of daily estradiol treatment (1 mg kg^-1^) during 8 weeks on body weight gain **(A)**, tibia length **(B)** and relation between uterus weight and tibia length **(C)**. Sham-operated (SHAM), ovariectomized (OVX), or estrogen-treated OVX (OVX+E) groups. Graph **(A)** represents mean ± SEM, **(B,C)** are bar graphs showing median ± interquartile ranges. ^∗^*P* < 0.01 vs. SHAM; ^#^*P* < 0.01 vs. OVX. Weight gain = body weight at the time of euthanasia – body weight on the day of surgery. The number of rats per group is indicated in the figure.

**Figure 2** shows that at the end of 8 weeks OVX animals presented greater weight gain than SHAM and OVX+E groups, demonstrated by one way-ANOVA followed by the Bonferroni post-test (*P* < 0.01, **Figure 2A**), with no differences among their sizes, evidenced by the tibia length (**Figure 2B**).

The efficacy of ovariectomy (OVX group) was determined according to Paigel et al. (2011), by the absence of ovarian tissue and marked atrophy of the uterus, as compared to the SHAM group (**Figure 2C**), while uterine hypertrophy was observed in the ovariectomized group treated with estradiol (OVX+E). These results reproduced data from our laboratory (Castardo-de-Paula et al., 2017).

### Hemodynamic and Autonomic Parameters Before Endotoxemia

SMT injection increased the blood pressure of all groups (**Table 1**, *P* ≤ 0.01), when compared to saline groups, being statistically significant in SHAM and OVX+E groups. Decreases in HR, by SMT injection, were statistically significant in OVX and OVX+E groups. Inhibition of iNOS with SMT significantly reduced the LF of SAP in OVX rats as compared to OVX rats receiving saline, and this reduction was maintained in estradiol treated animals compared to their OVX+E saline controls. SMT significantly increased BRS gain in OVX rats, while the OVX+E rats BRS increased were not significant (**Table 1**, *P* ≤ 0.05). In fact, the OVX and OVX+E groups presented higher HR responses in response to the SMT, corroborating this information.

**Table 1:** Hemodynamic variables, heart rate variability, systolic pressure variability, and baroreflex sensitivity just before LPS injection (time zero): Response to S-methylisothiourea sulfate (SMT).

	SHAM Saline (*n* = 9)	SHAM SMT (*n* = 8)	OVX Saline (*n* = 8)	OVX SMT (*n* = 9)	OVX+E Saline (*n* = 9)	OVX+E SMT (*n* = 8)
**HAEMODYNAMIC VARIABLES**						
MAP (mmHg)	104 (101–106)	119 (116–128)^∗∗^	107 (104–113)	124 (116–130)	111 (107–113)	135 (126–143)^∗∗^
HR (beats min^-1^)	348 ± 5.9	327 ± 15.0	362 ± 5.6	317 ± 6.0^∗^	382 ± 6.2	304 ± 12.6^∗∗∗^
**SBP**						
LF (mmHg^2^)	3.8 ± 0.5	2.2 ± 0.4	5.7 ± 0.8	2.5 ± 0.7^∗^	6.0 ± 1.2	1.9 ± 0.4^∗∗^
**PI**						
LFnu	37.9 ± 3.3	27.0 ± 4.8	38.4 ± 2.5	31.4 ± 3.2	38.7 ± 4.1	25.9 ± 3.7
HFnu	62.1 ± 3.3	73.0 ± 4.8	61.6 ± 2.5	68.6 ± 3.2	61.3 ± 4.1	74.1 ± 3.7
LF : HF ratio	0.7 ± 0.1	0.4 ± 0.1	0.7 ± 0.1	0.5 ± 0.1	0.8 ± 0.1	0.4 ± 0.1
**BRS**						
Total gain (ms mmHg^-1^)	1.5 (1.2–2.1)	1.2 (0.9–1.5)	0.9 (0.7–1.3)	2.3 (1.6–2.8)^∗^	1.3 (1.0–1.4)	3.8 (1.1–5.6)


*MAP, mean arterial pressure; HR, heart rate; SBP, systolic blood pressure; LF, low frequency; PI, pulse interval; HF, high frequency; BRS, baroreflex sensitivity. Data were obtained from the last 5 min of the recordings of arterial pressure pre-LPS injection. MAP and BRS gain were analyzed by Kruskal–Wallis test and are presented as median (interquartile range values); other data were analyzed by ANOVA and are represented as mean ± SEM. ^∗^Significant different from respective saline group: ^∗^P ≤ 0.05; ^∗∗^P ≤ 0.01; ^∗∗∗^P ≤ 0.001.*

### Hemodynamic and Autonomic Parameters After Endotoxemia

**FIGURE 3:**
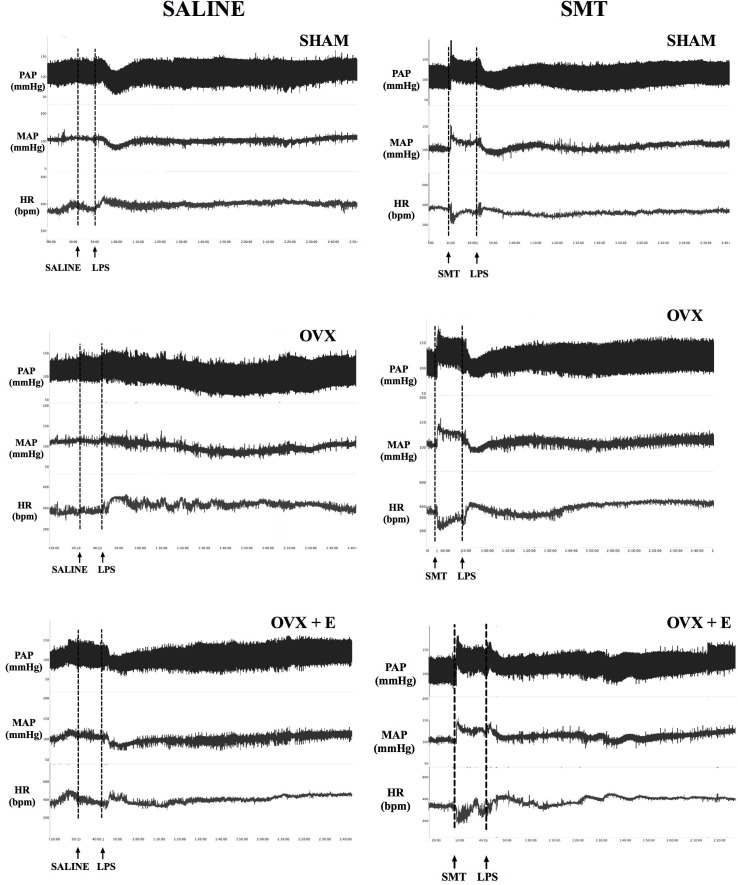
Typical recordings of pulsatile arterial pressure (PAP, mmHg), mean arterial pressure (MAP, mmHg) and heart rate (HR, bpm) illustrating the cardiovascular responses to intravenous LPS injection (5 mg kg^-1^) in non-anesthetized rats, preceded by saline or SMT (3 mg kg^-1^) injection, 8 weeks post sham-operation (SHAM), bilateral ovariectomy (OVX), or OVX plus daily estradiol treatment (OVX+E). Arrows indicate the time of injections.

**FIGURE 4:**
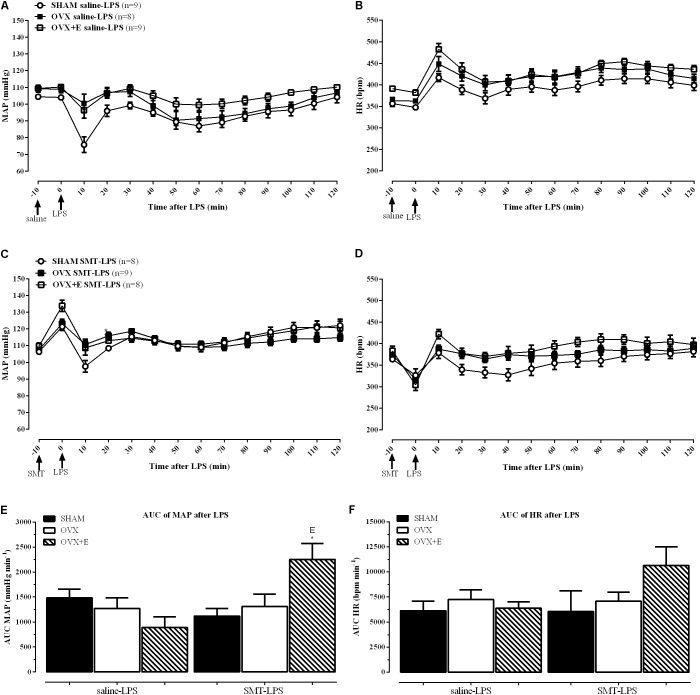
Time-course response to LPS (5 mg kg^-1^) of MAP **(A)** and HR **(B)** after saline (0.9%) injection, and MAP **(C)** and HR **(D)** after SMT (3 mg kg^-1^) injection. Mean ± SEM of five minutes before and the respective minute after LPS administration. Comparison of area under the curves (AUC) of MAP **(E)** and HR **(F)**. Differences of corresponding saline group: (S) SHAM, (O) OVX, and (E) OVX+E. Different from SHAM (^∗^) inside the treatment, (*P* < 0.05). The number of rats per group is indicated in the **(A,C)**.

Recordings showing the cardiovascular effects of SMT or saline injection before LPS in one SHAM, OVX and one OVX+E female rat are shown in **Figure 3**. Time-course of cardiovascular responses of saline or SMT pre-treated groups to LPS, 10 min before until 120 min after, can be observed in **Figure 4**. The AUC of MAP (**Figure 4E**) and HR (**Figure 4F**), time-course curves were performed and the MAP of OVX+E group pre-treated with SMT was greater than their respective SHAM SMT-LPS group (symbol^∗^) and OVX+E saline-LPS group (symbol E) (**Figure 4E**, *P* ≤ 0.05).

**FIGURE 5:**
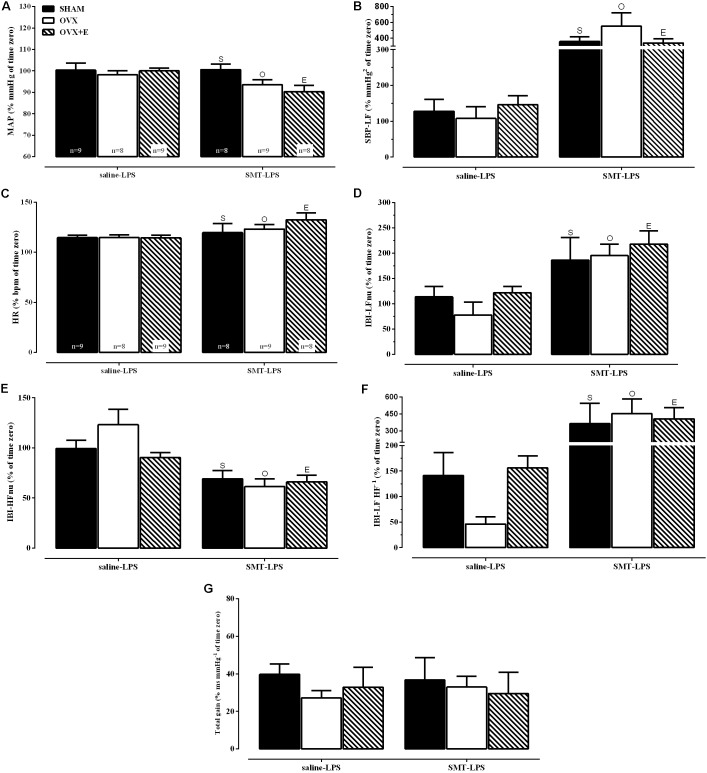
Cardiovascular and spectral hemodynamic data 2 h after LPS injection (5 mg kg^-1^) for sham-operated (SHAM), ovariectomized (OVX), or estrogen-treated OVX (OVX+E) groups treated with SMT (3 mg kg^-1^) or vehicle (saline 0.9%, 1 ml kg^-1^) 10 min before LPS. **(A)** Mean arterial pressure (MAP), **(B)** spectral density of systolic blood pressure in the low-frequency (SBP-LF), **(C)** heart rate (HR), **(D)** spectral densities of interbeat intervals (IBI) in the low-frequency (IBI-LFnu), **(E)** IBI in the high-frequency (IBI-HFnu), **(F)** LF HF^-1^ ratio (IBI-LF HF^-1^), **(G)** total baroreflex gain. Data are represented in % of time zero. Differences of corresponding saline group: (S) SHAM, (O) OVX and (E) OVX+E, *P* ≤ 0.05. Values are means ± S.E.M. The number of rats per group is indicated in the **(A,C)**.

In order to achieve homogeneity in the data distribution, the percentage (%) of changes in relation to time zero (100%, moment of LPS injection, **Table 1** and **Figures 3, 4**) was established for the cardiovascular and autonomic parameters, allowing the analysis of the effects of SMT injection in endotoxemia of SHAM, OVX and OVX+E female rats (**Figure 5**).

Groups pretreated with saline (saline-LPS, **Figure 5**) shows values of cardiovascular and autonomic parameters that served as controls for the study of the effects of iNOS inhibition by previous administration of SMT (3 mg kg^-1^), on LPS induced endotoxemia (SMT-LPS, **Figure 5**).

The analysis of variance (2-way ANOVA) indicated an effect of previous SMT injection, on the response to LPS, regardless of surgery or estradiol treatment, on % of MAP, on % of spectral density of systolic blood pressure (SBP) in the low frequency (SBP-LF), on % of HR and on % of HRV parameters (**Figures 5A–F**, *P* < 0.05). As described below.

MAP decreased in the endotoxemic females pre-treated with SMT injection **(Figure 5A**, *P* < 0.05) while the vascular sympathetic activity, measured by SBP-LF (**Figure 5B**, *P* < 0.01), increased in all groups, 2 h after LPS. **Figures 5C–F** shows the analysis of cardiac parameters 2 h after LPS of the animals pre-treated with saline or SMT. SMT injection increased the HR equivalently in all groups after LPS (2-way ANOVA: pre-LPS effect, *P* < 0.01, **Figure 5C**). The HRV analysis was performed to verify the influence of the autonomic nervous system (ANS) on HR. iNOS inhibition promoted equivalently increases in cardiac frequency domain parameters of LFnu and LF HF^-1^ (**Figures 5D,F**, respectively) in 2 h of endotoxemia, while the HFnu decreased (**Figure 5E**).

The analysis of the BRS (**Figure 5G**) revealed that pre-treatment with SMT did not alter the response of the females to the LPS, since they presented the same results observed with the saline treated animals, with reduction of more than 50% of total baroreflex gain.

### Plasmatic Biochemical Parameters After Endotoxemia

**FIGURE 6:**
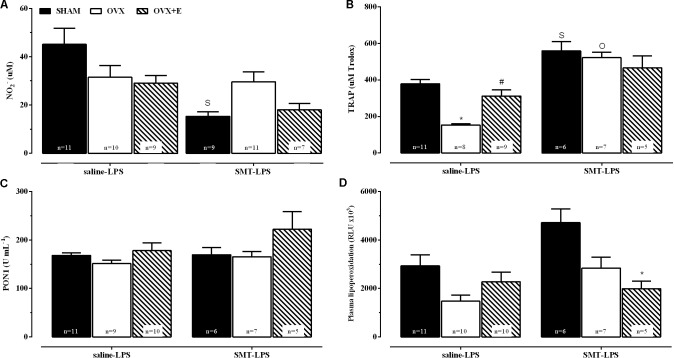
Plasma biochemical parameters, 2 h after LPS (5 mg kg^-1^), for sham-operated (SHAM), ovariectomized (OVX), or estrogen-treated OVX (OVX+E) groups pre-treated with SMT (3 mg kg^-1^) or vehicle (saline 0.9%, 1 ml kg^-1^) 10 min before LPS. **(A)** Nitrite levels, **(B)** total radical-trapping antioxidant parameter (TRAP), **(C)** paraoxonase 1 (PON1) activity and **(D)** lipoperoxidation. Values are means ± SEM. Differences of corresponding saline group: (S) SHAM, (O) OVX, and (E) OVX+E, *P* ≤ 0.05. ^∗^*P* < 0.01 vs. corresponding SHAM; ^#^*P* < 0.05 vs. corresponding OVX. The number of rats per group is indicated in the figure.

Plasma biochemical parameters 2 h after the induction of endotoxemia, in animals previously treated with saline or SMT are shown in **Figure 6** (saline-LPS and SMT-LPS, respectively).

The 2-way ANOVA compared the LPS-saline groups with the respective SMT-LPS in order to verify the effects of inhibition of iNOS in response of these parameters to endotoxemia. The SMT promoted a decrease in nitrite production in response to LPS in SHAM group, compared to SHAM-saline-LPS (**Figure 6A**, interaction between pre-LPS injection and surgery or estradiol treatment, *P* < 0.05). OVX rats pre-treated with saline presented a decrease in TRAP in relation to SHAM group, while estradiol treatment prevented this decrease (OVX+E group, **Figure 6B**, 2-way ANOVA, interaction between the two factors, *P* < 0.05). iNOS inhibition, before LPS, also increased the TRAP of the three experimental groups when compared to their respective saline-LPS, being statistically significant in the SHAM and OVX groups (**Figure 6B**, *P* ≤ 0.05, OVX+E *P* = 0.08).

Pre-treatment with SMT decreased the plasma LOOH of OVX+E endotoxemic rats, when compared to SHAM group (**Figure 6D**, 2-way ANOVA, effects of the two factors: pre-LPS and surgery or estradiol treatment, *P* < 0.05).

## Discussion

The present study demonstrated that the first minutes of exposure to SMT promote an increase in MAP that depends, at least in part, on estradiol, while the cardiac response and BRS are maintained by the ovarian hormones, as the OVX rats showed a significant reduction of HR and increased BRS that were not completely prevented in OVX+E rats. It also demonstrated that iNOS isoform preferentially appears to mediate the cardiac response of females to LPS independently of ovarian hormones, through sympathetic inhibition, since SMT increased this component of sympathovagal balance during endotoxemia.

The efficacy of hormonal deprivation, generated by OVX, and treatment with estradiol were confirmed by the results observed on body and uterine weights, confirming previous data from our laboratory (Castardo-de-Paula et al., 2017). These results are in agreement with the data presented by Paigel et al. (2011) and Ceylan-Isik et al. (2009), where the same time of evaluation of OVX and, in the case of Ceylan-Isik et al. (2009), the same treatment protocol with estradiol prevented the differences between groups in body and uterine weights.

The increase in MAP promoted by SMT injection, which was not statistically significant only in OVX rats, suggests initially that NO from the inducible pathway, in non-pathological states, has a lower participation in the blood pressure control of rats without ovaries. Since the OVX+E group had a statistically significant increase in MAP, estradiol could participate in the control of the MAP in females by association with iNOS. However, we have previously shown that inhibition of the constitutive pathway of NOS by L-NAME in non-pathological states promotes a markedly more intense hypertensive reaction than the action of SMT on MAP (Castardo-de-Paula et al., 2017). Indicating the inhibitory action of SMT on a NOS pathway that becomes more active in pathological states (iNOS pathway).

The literature shows a relation between the physiological activity of iNOS in females and estradiol levels, where iNOS participates in cardiac and vasodilator actions of NO (Nuedling et al., 1999; Zhu et al., 2002; Upmacis et al., 2011), justifying higher increases in MAP after its inhibition in female rats that have circulating estradiol (SHAM and OVX+E). It is important to note that, although the MAP responses seem to be initially independent of the sympathetic autonomic control (SBP-LF did not change or became smaller), previous data demonstrated increases of SBP-LF 2 h after SMT injection in female wistar rats (Castardo-de-Paula et al., 2017). Those data indicate that, physiologically, there is a shift for increased vascular sympathetic activity, suggesting inhibition by iNOS.

The HR response in OVX and OVX+E rats may reflect the increase in BRS observed in these groups. BRS was obtained by the sequence method of spontaneous baroreflex analysis, in which the gain increase reflects higher changes in the HR against spontaneous fluctuations of the blood pressure (Schwartz et al., 2013). In this case, the transient increase in sensitivity may have contributed to bradycardic responses to blood pressure increases. Ovarian hormones appear to work together to maintain the cardiac response and BRS, because OVX alterations were not prevented by treatment with estradiol. However, the absence of alteration 2 h after the SMT, observed in our previous work (Castardo-de-Paula et al., 2017), indicates that the change in HR is transitory, manifesting only at the beginning of the response.

Endotoxemic females pretreated with saline presented higher values of HR and lower BRS in relation to the moment of LPS administration. HR is known to increase under the effect of LPS (Annane et al., 1999; Mani et al., 2006) and, according to these authors, this reflects the balance between the cardiac autonomic activity and circulating hormones such as adrenaline and other vasoactive factors. Also, decreased BRS was observed in septic patients and may contribute to autonomic disturbances (Annane et al., 1999). Irigoyen et al. (2001) suggest that the reduction of BRS can trigger, by lower inhibition of vasomotor centers, increase of sympathetic activity. However, no changes were observed in the parameters of the cardiovascular spectral analysis, when the iNOS was active.

Females of the present study had blood pressure values equivalent to normal at the end of 2 h of endotoxemia. Such data differ from that observed by Mehanna et al. (2007), where males presented hypotension and tachycardia 2 h after LPS. In fact, Losonczy et al. (2000) observed that females are resistant to the development of endotoxin’s shock, confirming sexual dimorphism in the first few hours of endotoxemia.

Angele et al. (2014) discuss the observed differences in sepsis and present male sex as an independent risk factor for morbidity and mortality. However, these authors point estradiol as responsible for resistance to sepsis or endotoxemic stimulation with LPS. These differences related to estradiol were not observed in the cardiovascular parameters of the present study, as was not observed by Losonczy et al. (2000), where OVX did not alter hemodynamic parameters nor nitrite and nitrate levels after LPS. These authors argue that ovariectomy may not significantly alter the immune response but, the effects of treatment with estradiol on the response to LPS may be time dependent, since 12 h after endotoxemia NO production was lower in intact and estrogen-treated females (Losonczy et al., 2000).

After 2 h of endotoxemia, ovariectomy reduced TRAP and treatment with estradiol prevented this change. The reduction of the plasma antioxidant capacity due to ovarian withdrawal and its prevention by treatment with estradiol has been observed previously (Hernandez et al., 2000), where it is argued that the reduction of this capacity would reflect an imbalance in the redox state with pre-dominance of oxidative processes. In fact, the pro-oxidant profile of OVX rats has already been demonstrated, including in important tissues for cardiovascular function (Ceravolo et al., 2013; Claudio et al., 2014). We have previously demonstrated that OVX rats, without LPS, tend to reduce TRAP (Castardo-de-Paula et al., 2017), and the endotoxemic state seems to have intensified this characteristic, probably due to the consumption of the antioxidant reserves when generating more reactive species. In fact, White et al. (2010) state that diseases lead to the reduction of essential cofactors to NO-generating activity by NOS and, in the absence of these, NOS become decoupled from the ability to generate NO and generate superoxide anion, a highly reactive oxygen specie.

Data suggest that iNOS has a physiological function independent of ovarian hormones and important for cardiovascular system. Chan et al. (2001) describe a tonic activity of iNOS in the central nervous system of males, inhibiting sympathetic efflux by RVLM. We demonstrated in a previous work the pre-dominance of sympathetic ANS activity after the selective inhibition of iNOS in females (Castardo-de-Paula et al., 2017). In the present study the role of iNOS in the response of females to 2 h of LPS endotoxemia was investigated by previous administration of SMT (3 mg kg^-1^), an iNOS selective inhibitor (Szabo et al., 1994).

Interestingly, blood pressure was reduced in the female rats treated with SMT. Previous data from our laboratory demonstrated increased MAP 2 h after SMT injection (Castardo-de-Paula et al., 2017), indicating a response related to endotoxin.

Injection of LPS promotes a systemic inflammatory response accompanied by the release of important cytokines to hypotension and shock, such as TNFa, IL-6, and IL-1. It is possible that the uncoupled iNOS, acting as a generator of reactive oxygen species vasoconstrictors (White et al., 2010), would counterbalance the hypotensive effect of pro-inflammatory cytokines. Therefore, inhibition of iNOS with SMT resulted in a reduction in blood pressure in endotoxemic rats. Inhibition of iNOS also promoted vascular and cardiac sympathetic activation, and increased TRAP in endotoxemic females. Among several pro-inflammatory mediators, LPS itself is a stimulus to iNOS activity (Lirk et al., 2002), while sympathetic ANS has a protective function against sepsis, favoring the maintenance of cardiac output (Vitecek et al., 2012) and containing the immune response (Martelli et al., 2014).

Mani et al. (2006) observed that knockout mice for iNOS had higher cardiac sympathetic activity, suggesting a tonic inhibitory role for iNOS in the heart. However, unlike the results observed by us in females, the authors observed that cardiac sympathetic modulation decreases during LPS endotoxemia, regardless of the presence of iNOS. These data suggest that the female sex would be a determinant of this sympathetic activity after SMT in endotoxemia.

Losonczy et al. (2000) observed no shock at 2–4 h after LPS in Sprague-Dawley female rats when compared to males, with no influence of prior castration. Investigating this protective role of female sex against hypotension and shock in sepsis, Wang et al. (2000) observed decrease of noradrenaline levels and increase of NOS activity after LPS, while selective inhibition of iNOS prevented the decrease of noradrenaline. Therefore, iNOS seems to favor the worsening of the sepsis by reducing the sympathetic activity.

Pacher et al. (2007) discuss the link between NO from iNOS and increased superoxide production in peroxynitrite generation, pointing also studies on the protection obtained with the selective inhibition of iNOS and its similarity to the use of antioxidants (Pacher et al., 2007). Such observations suggest a pro-oxidant action of iNOS in pathological states, which is reinforced by the increase of TRAP in all animals after inhibition of iNOS with SMT in the present work.

We have already observed that the inhibition of iNOS drives to a pre-dominance of sympathetic ANS (Castardo-de-Paula et al., 2017), however there are few data available on the relation between iNOS and sympathetic ANS in females in sepsis, being this the first observation of such factors in female Wistar rats, in the first hours of LPS endotoxemia. Therefore, we can affirm that the previous inhibition of iNOS generated a favorable cardiovascular and biochemical profile in females, in 2 h of endotoxemia, which involves the activation of the sympathetic ANS.

OVX rats, in the face of inflammatory challenges, express more iNOS than SHAM rats (Crisafulli et al., 2009; Ma and Bai, 2012; Sakanashi et al., 2013; Hassouna et al., 2014), and estradiol is a known stimulator of NO synthesis, including the increase of iNOS expression and activity (Nuedling et al., 1999; Zhu et al., 2002). In addition, progesterone is able to reduce iNOS activity in LPS-stimulated cells (Su et al., 2007; Menzies et al., 2011), suggesting that the lower plasma levels of nitrite observed in the SHAM group may be the result of a synergism between iNOS inhibition by SMT and the reduction of the activity of this enzyme by circulating progesterone of these animals. However, it is important to note that differences in plasma levels of nitrite do not necessarily reflect the levels of NO in tissues, since a reduction in one of these parameters can be observed at the same time as increase in the other, as observed by Panis et al. (2011) in plasma nitrite and cardiac tissue levels.

Sharma et al. (2008) reported that lipid peroxidation could increase by low antioxidant levels, increased substrates for oxidation, and the presence of pro-oxidants in plasma. We could suggest that the reduction of LOOH levels in SMT pre-treated OVX+E rats occurred by the increase of antioxidant levels, the decrease of the substrates for oxidation, or by the decrease of the levels of pro-oxidants. In fact, all endotoxemic females, pre-treated with iNOS inhibitor, showed an increase in total antioxidant capacity, and the similarity between the protection obtained with the inhibition of iNOS and the use of antioxidants was previously mentioned (Pacher et al., 2007). Suggesting that treatment with estradiol, in the absence of iNOS activity, creates a more favorable environment to the body’s response in 2 h of endotoxemia, than the set of ovarian hormones. Since estradiol itself is discussed as an antioxidant (Hernandez et al., 2000), this feature may have contributed to the reduction of LOOHs compared to SHAM group. To date, this is the first observation of these aspects in endotoxemic rats, involving or not the presence of estrogen.

The main limitation of our study is that SMT, although it has greater affinity or binding capacity for iNOS, is not a specific inhibitor of this, and there may be a minor inhibitory effect on the other isoforms of NOS. However, endotoxemia and LPS are well-established stimuli of iNOS, which is therefore the pre-dominant isoform in the experimental model of this article.

Literature point the male sex as an independent risk factor for morbidity and mortality in sepsis. This study demonstrates that females respond to the first few hours of endotoxemia with MAP maintenance and tachycardia. The mentioned facts for the preferential inhibition of iNOS, besides indicating that progesterone, or the set of ovarian hormones, may have an inhibitory action on the isoform, allow us to conclude, for the first time in the literature, that iNOS is responsible for the sympathetic inhibition and the consumption of the antioxidant reserves apart of estradiol levels, which may lead to the aggravation of endotoxemia and cardiovascular collapse in females.

## Data Availability

The raw data supporting the conclusions of this manuscript will be made available by the authors, without undue reservation, to any qualified researcher.

## Author Contributions

JC-d-P contributed to conception and design of the work, acquisition of the data, analysis and interpretation of the data, statistical analysis, and draft the manuscript. BdC, LdJ, and EA contributed to acquisition and analysis of the data. NZ, CdF, and LH contributed to acquisition of the data, analysis and interpretation of the data. PP-F and DB contributed to analysis and interpretation of the data. MM-P contributed to conception and design of the work, interpretation of the data, and draft the manuscript.

## Conflict of Interest Statement

The authors declare that the research was conducted in the absence of any commercial or financial relationships that could be construed as a potential conflict of interest.

## References

[B1] Angele M. K. Pratschke S. Hubbard W. J. Chaudry I. H. (2014). Gender differences in sepsis: cardiovascular and immunological aspects. *Virulence* 5 12–19. 10.4161/viru.26982 24193307 PMC3916365

[B2] Annane D. Trabold F. Sharshar T. Jarrin I. Blanc A. S. Raphael J. C. (1999). Inappropriate sympathetic activation at onset of septic shock: a spectral analysis approach. *Am. J. Respir. Crit. Care Med.* 160 458–465. 10.1164/ajrccm.160.2.9810073 10430714

[B3] Ariza D. Sisdeli L. Crestani C. C. Fazan R. Martins-Pinge M. C. (2015). Dysautonomias in Parkinson’s disease: cardiovascular changes and autonomic modulation in conscious rats after infusion of bilateral 6-OHDA in substantia nigra. *Am. J. Physiol. Heart Circ. Physiol.* 308 H250–H257. 10.1152/ajpheart.00406.2014 25416189

[B4] Bone R. C. (1992). Toward an epidemiology and natural history of SIRS (systemic inflammatory response syndrome). *JAMA* 268 3452–3455. 10.1001/jama.1992.03490240060037 1460735

[B5] Campos C. Casali K. R. Baraldi D. Conzatti A. Araujo A. S. Khaper N. (2014). Efficacy of a low dose of estrogen on antioxidant defenses and heart rate variability. *Oxid. Med. Cell. Longev.* 2014:218749. 10.1155/2014/218749 24738017 PMC3964890

[B6] Castardo-de-Paula J. C. de Campos B. H. Amorim E. D. T. da Silva R. V. de Farias C. C. Higachi L. (2017). Cardiovascular risk and the effect of nitric oxide synthase inhibition in female rats: the role of estrogen. *Exp. Gerontol.* 97 38–48. 10.1016/j.exger.2017.07.016 28757113

[B7] Cauwels A. (2007). Nitric oxide in shock. *Kidney Int.* 72 557–565. 10.1038/sj.ki.5002340 17538569

[B8] Ceravolo G. S. Filgueira F. P. Costa T. J. Lobato N. S. Chignalia A. Z. Araujo P. X. (2013). Conjugated equine estrogen treatment corrected the exacerbated aorta oxidative stress in ovariectomized spontaneously hypertensive rats. *Steroids* 78 341–346. 10.1016/j.steroids.2012.11.018 23261957

[B9] Ceylan-Isik A. F. Erdogan-Tulmac O. B. Ari N. Ozansoy G. Ren J. (2009). Effect of 17beta-oestradiol replacement on vascular responsiveness in ovariectomized diabetic rats. *Clin. Exp. Pharmacol. Physiol.* 36 e65–e71. 10.1111/j.1440-1681.2009.05255.x 19566816

[B10] Chan S. H. Wang L. L. Wang S. H. Chan J. Y. (2001). Differential cardiovascular responses to blockade of nNOS or iNOS in rostral ventrolateral medulla of the rat. *Br. J. Pharmacol.* 133 606–614. 10.1038/sj.bjp.0704105 11399678 PMC1572812

[B11] Claudio E. R. Endlich P. W. Santos R. L. Moyses M. R. Bissoli N. S. Gouvea S. A. (2014). Effects of chronic swimming training and oestrogen therapy on coronary vascular reactivity and expression of antioxidant enzymes in ovariectomized rats. *PLoS One* 8:e64806. 10.1371/journal.pone.0064806 23755145 PMC3670897

[B12] Crisafulli C. Bruscoli S. Esposito E. Mazzon E. Di Paola R. Genovese T. (2009). PPAR-alpha contributes to the anti-inflammatory activity of 17beta-estradiol. *J. Pharmacol. Exp. Ther.* 331 796–807. 10.1124/jpet.109.156646 19755663

[B13] da Cunha N. V. Pinge-Filho P. Panis C. Silva B. R. Pernomian L. Grando M. D. (2014). Decreased endothelial nitric oxide, systemic oxidative stress, and increased sympathetic modulation contribute to hypertension in obese rats. *Am. J. Physiol. Heart Circ. Physiol.* 306 H1472–H1480. 10.1152/ajpheart.00520.2013 24633548

[B14] Dutra S. G. Pereira A. P. Tezini G. C. Mazon J. H. Martins-Pinge M. C. Souza H. C. (2013). Cardiac autonomic modulation is determined by gender and is independent of aerobic physical capacity in healthy subjects. *PLoS One* 8:e77092. 10.1371/journal.pone.0077092 24098577 PMC3789672

[B15] Gore A. C. Oung T. Woller M. J. (2002). Age-related changes in hypothalamic gonadotropin-releasing hormone and N-methyl-D-aspartate receptor gene expression, and their regulation by oestrogen, in the female rat. *J. Neuroendocrinol.* 14 300–309. 10.1046/j.1365-2826.2002.00777.x 11963827

[B16] Hassouna A. Obaia E. Marzouk S. Rateb M. Haidara M. (2014). The role of sex hormones in induced-systemic inflammation in female albino rats. *Acta Physiol. Hung.* 101 112–127. 10.1556/APhysiol.101.2014.1.12 24631798

[B17] Hernandez I. Delgado J. L. Diaz J. Quesada T. Teruel M. J. Llanos M. C. (2000). 17beta-estradiol prevents oxidative stress and decreases blood pressure in ovariectomized rats. *Am. J. Physiol. Regul. Integr. Comp. Physiol.* 279 R1599–R1605. 10.1152/ajpregu.2000.279.5.R1599 11049841

[B18] Huang J. Wang Y. Jiang D. Zhou J. Huang X. (2010). The sympathetic-vagal balance against endotoxemia. *J. Neural Transm.* 117 729–735. 10.1007/s00702-010-0407-6 20458507

[B19] Irigoyen M. C. Consolim-Colombo F. M. Krieger E. M. (2001). Controle cardiovascular: regulação reflexa e papel do sistema nervoso simpático. *Rev. Bras. Hiperten.* 8 55–62.

[B20] Kauser K. Sonnenberg D. Tse J. Rubanyi G. M. (1997). 17 beta-Estradiol attenuates endotoxin-induced excessive nitric oxide production in ovariectomized rats in vivo. *Am. J. Physiol.* 273(1 Pt 2), H506–H509. 10.1152/ajpheart.1997.273.1.H506 9249525

[B21] Knoferl M. W. Angele M. K. Diodato M. D. Schwacha M. G. Ayala A. Cioffi W. G. (2002). Female sex hormones regulate macrophage function after trauma-hemorrhage and prevent increased death rate from subsequent sepsis. *Ann. Surg.* 235 105–112. 10.1097/00000658-200201000-00014 11753049 PMC1422402

[B22] Kuo T. B. Lai C. T. Hsu F. C. Tseng Y. J. Li J. Y. Shieh K. R. (2010). Cardiac neural regulation oscillates with the estrous cycle in freely moving female rats: the role of endogenous estrogens. *Endocrinology* 151 2613–2621. 10.1210/en.2009-1410 20392827

[B23] Lirk P. Hoffmann G. Rieder J. (2002). Inducible nitric oxide synthase–time for reappraisal. *Curr. Drug Targets Inflamm. Allergy* 1 89–108. 10.2174/1568010023344913 14561209

[B24] Losonczy G. Kriston T. Szabo A. Muller V. Harvey J. Hamar P. (2000). Male gender predisposes to development of endotoxic shock in the rat. *Cardiovasc. Res.* 47 183–191. 10.1016/S0008-6363(00)00075-4 10869545 PMC2756823

[B25] Ma Z. Bai L. (2012). The anti-inflammatory effect of Z-Ligustilide in experimental ovariectomized osteopenic rats. *Inflammation* 35 1793–1797. 10.1007/s10753-012-9499-5 22760256

[B26] Mani A. R. Ollosson R. Mani Y. Ippolito S. Moore K. P. (2006). Heart rate dynamics in iNOS knockout mice. *Life Sci.* 79 1593–1599. 10.1016/j.lfs.2006.05.014 16790251

[B27] Martelli D. Yao S. T. McKinley M. J. McAllen R. M. (2014). Reflex control of inflammation by sympathetic nerves, not the vagus. *J. Physiol.* 592 1677–1686. 10.1113/jphysiol.2013.268573 24421357 PMC3979618

[B28] Mehanna A. Vitorino D. C. Panis C. Blanco E. E. Pinge-Filho P. Martins-Pinge M. C. (2007). Cardiovascular and pulmonary effects of NOS inhibition in endotoxemic conscious rats subjected to swimming training. *Life Sci.* 81 1301–1308. 10.1016/j.lfs.2007.09.006 17916368

[B29] Menzies F. M. Henriquez F. L. Alexander J. Roberts C. W. (2011). Selective inhibition and augmentation of alternative macrophage activation by progesterone. *Immunology* 134 281–291. 10.1111/j.1365-2567.2011.03488.x 21977998 PMC3209568

[B30] Nuedling S. Kahlert S. Loebbert K. Doevendans P. A. Meyer R. Vetter H. (1999). 17 Beta-estradiol stimulates expression of endothelial and inducible NO synthase in rat myocardium in-vitro and in-vivo. *Cardiovasc. Res.* 43 666–674. 10.1016/S0008-6363(99)00093-0 10690338

[B31] Pacher P. Beckman J. S. Liaudet L. (2007). Nitric oxide and peroxynitrite in health and disease. *Physiol. Rev.* 87 315–424. 10.1152/physrev.00029.2006 17237348 PMC2248324

[B32] Paigel A. S. Ribeiro R. F. Jr. Fernandes A. A. Targueta G. P. Vassallo D. V. Stefanon I. (2011). Myocardial contractility is preserved early but reduced late after ovariectomy in young female rats. *Reprod. Biol. Endocrinol.* 9:54. 10.1186/1477-7827-9-54 21513549 PMC3107166

[B33] Panis C. Mazzuco T. L. Costa C. Z. Victorino V. J. Tatakihara V. L. Yamauchi L. M. (2011). *Trypanosoma cruzi*: effect of the absence of 5-lipoxygenase (5-LO)-derived leukotrienes on levels of cytokines, nitric oxide and iNOS expression in cardiac tissue in the acute phase of infection in mice. *Exp. Parasitol.* 127 58–65. 10.1016/j.exppara.2010.06.030 20599987

[B34] Petrofsky J. S. Lohman E. Lohman T. (2009). A device to evaluate motor and autonomic impairment. *Med. Eng. Phys.* 31 705–712. 10.1016/j.medengphy.2009.01.007 19251462

[B35] Sakanashi M. Matsuzaki T. Noguchi K. Nakasone J. Sakanashi M. Uchida T. (2013). Long-term treatment with san’o-shashin-to, a kampo medicine, markedly ameliorates cardiac ischemia-reperfusion injury in ovariectomized rats via the redox-dependent mechanism. *Circ. J.* 77 1827–1837. 10.1253/circj.CJ-12-1434 23615023

[B36] Schwartz C. E. Medow M. S. Messer Z. Stewart J. M. (2013). Spontaneous fluctuation indices of the cardiovagal baroreflex accurately measure the baroreflex sensitivity at the operating point during upright tilt. *Am. J. Physiol. Regul. Integr. Comp. Physiol.* 304 R1107–R1113. 10.1152/ajpregu.00559.2012 23576616 PMC3680794

[B37] Sharma A. Kaur P. Kumar B. Prabhakar S. Gill K. D. (2008). Plasma lipid peroxidation and antioxidant status of Parkinson’s disease patients in the Indian population. *Parkinsonism Relat. Disord.* 14 52–57. 10.1016/j.parkreldis.2007.06.009 18032086

[B38] Straub R. H. (2007). The complex role of estrogens in inflammation. *Endocr. Rev.* 28 521–574. 10.1210/er.2007-0001 17640948

[B39] Su C. F. Yang F. L. Chen H. I. (2007). Inhibition of inducible nitric oxide synthase attenuates acute endotoxin-induced lung injury in rats. *Clin. Exp. Pharmacol. Physiol.* 34 339–346. 10.1111/j.1440-1681.2007.04553.x 17324147

[B40] Szabo C. Southan G. J. Thiemermann C. (1994). Beneficial effects and improved survival in rodent models of septic shock with S-methylisothiourea sulfate, a potent and selective inhibitor of inducible nitric oxide synthase. *Proc. Natl. Acad. Sci. U.S.A.* 91 12472–12476. 10.1073/pnas.91.26.12472 7528923 PMC45460

[B41] Upmacis R. K. Shen H. Benguigui L. E. S. Lamon B. D. Deeb R. S. Hajjar K. A. (2011). Inducible nitric oxide synthase provides protection against injury-induced thrombosis in female mice. *Am. J. Physiol. Heart Circ. Physiol.* 301 H617–H624. 10.1152/ajpheart.00667.2010 21602468 PMC3154673

[B42] Vitecek J. Lojek A. Valacchi G. Kubala L. (2012). Arginine-based inhibitors of nitric oxide synthase: therapeutic potential and challenges. *Med. Inflamm.* 2012:318087. 10.1155/2012/318087 22988346 PMC3441039

[B43] Voltera A. F. Cesaretti M. L. Ginoza M. Kohlmann O. Jr. (2008). Effects of neuroendocrine obesity induction on systemic hemodynamics and left ventricular function of normotensive rats. *Arq. Bras. Endocrinol. Metabol.* 52 47–54. 10.1590/S0004-27302008000100008 18345396

[B44] Wang Y. Steinsland O. S. Nelson S. H. (2000). A role for nitric oxide in endotoxin-induced depletion of the peripheral catecholamine stores. *Shock* 13 145–151. 10.1097/00024382-200013020-00009 10670845

[B45] White R. E. Gerrity R. Barman S. A. Han G. (2010). Estrogen and oxidative stress: a novel mechanism that may increase the risk for cardiovascular disease in women. *Steroids* 75 788–793. 10.1016/j.steroids.2009.12.007 20060403 PMC2891201

[B46] Zhu Y. Bian Z. Lu P. Karas R. H. Bao L. Cox D. (2002). Abnormal vascular function and hypertension in mice deficient in estrogen receptor β. *Science* 295 505–508. 10.1126/science.1065250 11799247

